# Supplementary material for: Hot spot prediction in protein-protein interactions by an ensemble system
Source: BMC Syst Biol. 2018 Dec 31;12(Suppl 9):132. doi: 10.1186/s12918-018-0665-8 (PMC6311905; doi:10.1186/s12918-018-0665-8)
Supplement: Supplementary file 2 — All relevant MATLAB code. A simple Matlab implement of our predictor. (ZIP 19,742 kb) [file 12918_2018_665_MOESM2_ESM.zip › Additional file 2/Read ASA/ASA website/dbMPIKT/4UWQA.html]

 NetSurfP - Protein Surface Accessibility and Secondary Structure Predictions

  

|  |  |  |
| --- | --- | --- |
|  |  | NetSurfP - Protein Surface Accessibility and Secondary Structure Predictions  Technical University of Denmark |

  


---

```
# For publication of results, please cite:
# A generic method for assignment of reliability scores applied to solvent accessibility predictions.
# Bent Petersen, Thomas Nordahl Petersen, Pernille Andersen, Morten Nielsen and Claus Lundegaard
# BMC Structural Biology 2009, 9:51 doi:10.1186/1472-6807-9-51
#
# Column 1: Class assignment - B for buried or E for Exposed - Threshold: 25% exposure, but not based on RSA
# Column 2: Amino acid
# Column 3: Sequence name
# Column 4: Amino acid number
# Column 5: Relative Surface Accessibility - RSA
# Column 6: Absolute Surface Accessibility
# Column 7: Z-fit score for RSA prediction
# Column 8: Probability for Alpha-Helix
# Column 9: Probability for Beta-strand
# Column 10: Probability for Coil
E M  4UWQA                  1    0.788 157.679  -1.622   0.003   0.003   0.994
E A  4UWQA                  2    0.436  48.047  -1.187   0.018   0.047   0.935
E S  4UWQA                  3    0.506  59.315  -0.690   0.113   0.087   0.800
B W  4UWQA                  4    0.292  70.274  -0.920   0.184   0.043   0.773
E S  4UWQA                  5    0.384  45.028  -1.752   0.257   0.016   0.727
E H  4UWQA                  6    0.325  59.099   0.565   0.339   0.016   0.645
E P  4UWQA                  7    0.339  48.062  -0.747   0.522   0.016   0.462
E Q  4UWQA                  8    0.429  76.530  -0.203   0.717   0.014   0.269
E F  4UWQA                  9    0.423  84.816  -1.177   0.717   0.014   0.269
E E  4UWQA                 10    0.408  71.278  -0.863   0.622   0.015   0.363
E K  4UWQA                 11    0.454  93.388  -0.981   0.622   0.015   0.363
E G  4UWQA                 12    0.445  35.029  -0.840   0.502   0.002   0.495
B A  4UWQA                 13    0.221  24.343  -0.927   0.339   0.016   0.645
E L  4UWQA                 14    0.446  81.754   0.075   0.257   0.016   0.727
E E  4UWQA                 15    0.601 104.977   0.056   0.257   0.016   0.727
E D  4UWQA                 16    0.541  77.929   0.123   0.181   0.016   0.803
E P  4UWQA                 17    0.340  48.203  -1.178   0.181   0.016   0.803
E R  4UWQA                 18    0.501 114.637   0.095   0.115   0.016   0.868
E S  4UWQA                 19    0.467  54.709   0.081   0.115   0.016   0.868
B L  4UWQA                 20    0.148  27.062  -0.801   0.181   0.016   0.803
B Y  4UWQA                 21    0.251  53.553  -0.354   0.113   0.043   0.844
E D  4UWQA                 22    0.601  86.575  -0.095   0.053   0.043   0.903
B L  4UWQA                 23    0.141  25.835   0.293   0.053   0.043   0.903
E P  4UWQA                 24    0.503  71.376   0.410   0.053   0.043   0.903
E P  4UWQA                 25    0.482  68.467  -0.304   0.052   0.084   0.864
B Y  4UWQA                 26    0.268  57.229   0.001   0.056   0.142   0.802
B G  4UWQA                 27    0.160  12.561  -1.209   0.019   0.141   0.840
E D  4UWQA                 28    0.402  57.885   0.753   0.019   0.141   0.840
B A  4UWQA                 29    0.030   3.339   0.357   0.023   0.655   0.322
B T  4UWQA                 30    0.210  29.085   0.802   0.018   0.846   0.136
B L  4UWQA                 31    0.035   6.390   0.450   0.011   0.918   0.071
B L  4UWQA                 32    0.025   4.559   0.847   0.018   0.846   0.136
B Y  4UWQA                 33    0.070  14.959  -0.025   0.018   0.846   0.136
B F  4UWQA                 34    0.034   6.924   0.075   0.023   0.655   0.322
B S  4UWQA                 35    0.028   3.305   0.289   0.069   0.386   0.545
B D  4UWQA                 36    0.057   8.170  -0.576   0.125   0.227   0.648
B L  4UWQA                 37    0.052   9.466  -0.378   0.191   0.086   0.723
B H  4UWQA                 38    0.067  12.096   0.144   0.268   0.043   0.689
B G  4UWQA                 39    0.059   4.628  -1.274   0.184   0.043   0.773
B Q  4UWQA                 40    0.201  35.934  -0.339   0.184   0.043   0.773
B A  4UWQA                 41    0.105  11.582  -0.897   0.113   0.087   0.800
E F  4UWQA                 42    0.275  55.112   0.173   0.052   0.084   0.864
B P  4UWQA                 43    0.180  25.542  -1.216   0.019   0.141   0.840
B H  4UWQA                 44    0.192  34.925  -0.882   0.066   0.296   0.638
B Y  4UWQA                 45    0.184  39.406  -1.009   0.069   0.386   0.545
B F  4UWQA                 46    0.116  23.261  -1.372   0.069   0.386   0.545
B M  4UWQA                 47    0.223  44.602  -0.799   0.066   0.296   0.638
B E  4UWQA                 48    0.259  45.317  -0.503   0.064   0.216   0.721
B P  4UWQA                 49    0.161  22.860  -0.791   0.056   0.142   0.802
B P  4UWQA                 50    0.202  28.678  -1.686   0.056   0.142   0.802
B N  4UWQA                 51    0.215  31.403  -1.081   0.125   0.227   0.648
B L  4UWQA                 52    0.253  46.306  -1.712   0.125   0.227   0.648
B I  4UWQA                 53    0.142  26.251  -0.953   0.056   0.142   0.802
B A  4UWQA                 54    0.200  22.018  -2.172   0.056   0.142   0.802
B P  4UWQA                 55    0.229  32.453  -0.671   0.118   0.150   0.732
E K  4UWQA                 56    0.296  60.908  -0.810   0.118   0.150   0.732
E P  4UWQA                 57    0.335  47.607  -1.128   0.199   0.152   0.649
B L  4UWQA                 58    0.208  38.103  -1.580   0.199   0.152   0.649
E M  4UWQA                 59    0.339  67.834  -0.606   0.113   0.087   0.800
E G  4UWQA                 60    0.400  31.504  -1.857   0.052   0.084   0.864
B R  4UWQA                 61    0.278  63.754   0.049   0.053   0.043   0.903
B P  4UWQA                 62    0.237  33.644  -0.431   0.113   0.043   0.844
B G  4UWQA                 63    0.155  12.206  -1.313   0.199   0.152   0.649
B Y  4UWQA                 64    0.192  40.924  -0.569   0.216   0.235   0.548
B L  4UWQA                 65    0.103  18.914  -1.039   0.216   0.235   0.548
B T  4UWQA                 66    0.107  14.813  -0.685   0.307   0.165   0.527
B G  4UWQA                 67    0.100   7.909  -0.776   0.354   0.048   0.598
E E  4UWQA                 68    0.395  68.937  -0.000   0.522   0.016   0.462
E A  4UWQA                 69    0.430  47.397  -0.591   0.522   0.016   0.462
B I  4UWQA                 70    0.156  28.934  -0.506   0.622   0.015   0.363
B L  4UWQA                 71    0.221  40.428  -0.543   0.622   0.015   0.363
E R  4UWQA                 72    0.618 141.614   0.305   0.622   0.015   0.363
E Y  4UWQA                 73    0.382  81.569   0.191   0.561   0.047   0.393
B Y  4UWQA                 74    0.199  42.441  -0.316   0.354   0.048   0.598
E G  4UWQA                 75    0.569  44.820  -0.684   0.115   0.016   0.868
B V  4UWQA                 76    0.229  35.136  -0.732   0.058   0.017   0.925
E E  4UWQA                 77    0.672 117.346   0.735   0.018   0.019   0.964
E R  4UWQA                 78    0.619 141.682  -0.821   0.018   0.019   0.964
E G  4UWQA                 79    0.652  51.297  -0.819   0.018   0.019   0.964
E T  4UWQA                 80    0.440  61.014   0.164   0.018   0.047   0.935
B P  4UWQA                 81    0.185  26.195   0.617   0.005   0.336   0.660
E L  4UWQA                 82    0.404  74.064   2.237   0.003   0.718   0.279
B A  4UWQA                 83    0.019   2.127   1.429   0.001   0.900   0.099
B Y  4UWQA                 84    0.146  31.157   1.638   0.001   0.959   0.040
B L  4UWQA                 85    0.019   3.570   1.365   0.001   0.959   0.040
B L  4UWQA                 86    0.032   5.914   0.737   0.001   0.959   0.040
B S  4UWQA                 87    0.033   3.914  -0.179   0.011   0.918   0.071
B Y  4UWQA                 88    0.036   7.779   0.285   0.021   0.756   0.223
B V  4UWQA                 89    0.039   6.056  -0.391   0.066   0.296   0.638
B D  4UWQA                 90    0.070  10.101  -0.643   0.113   0.087   0.800
B F  4UWQA                 91    0.055  10.958   0.155   0.199   0.152   0.649
B V  4UWQA                 92    0.058   8.838   0.155   0.278   0.093   0.628
B E  4UWQA                 93    0.070  12.212  -1.585   0.278   0.093   0.628
B L  4UWQA                 94    0.177  32.464   0.430   0.278   0.093   0.628
B A  4UWQA                 95    0.037   4.099  -0.903   0.278   0.093   0.628
E R  4UWQA                 96    0.324  74.150   0.492   0.184   0.043   0.773
E T  4UWQA                 97    0.592  82.069  -0.413   0.113   0.043   0.844
E F  4UWQA                 98    0.461  92.442  -1.632   0.058   0.017   0.925
E G  4UWQA                 99    0.578  45.504  -1.148   0.058   0.017   0.925
E P  4UWQA                100    0.452  64.125  -0.543   0.053   0.043   0.903
E I  4UWQA                101    0.304  56.314  -1.684   0.113   0.043   0.844
B G  4UWQA                102    0.192  15.142  -2.062   0.053   0.043   0.903
B G  4UWQA                103    0.160  12.592  -2.205   0.053   0.043   0.903
B M  4UWQA                104    0.093  18.549   0.584   0.879   0.010   0.111
B G  4UWQA                105    0.126   9.916  -1.415   0.938   0.007   0.055
B A  4UWQA                106    0.203  22.327   0.755   0.970   0.001   0.030
B L  4UWQA                107    0.029   5.255   0.485   0.970   0.001   0.030
B T  4UWQA                108    0.079  10.916   0.338   0.988   0.000   0.012
E A  4UWQA                109    0.261  28.817   0.709   0.988   0.000   0.012
B L  4UWQA                110    0.085  15.509   0.941   0.988   0.000   0.012
B I  4UWQA                111    0.021   3.922   1.211   0.988   0.000   0.012
E R  4UWQA                112    0.388  88.760   1.915   0.970   0.001   0.030
E D  4UWQA                113    0.516  74.298   1.543   0.970   0.001   0.030
B Q  4UWQA                114    0.079  14.181   0.848   0.970   0.001   0.030
B K  4UWQA                115    0.209  43.032   1.074   0.923   0.002   0.076
E A  4UWQA                116    0.622  68.533   1.013   0.923   0.002   0.076
E R  4UWQA                117    0.499 114.294   1.386   0.858   0.002   0.139
B V  4UWQA                118    0.116  17.891  -0.323   0.782   0.003   0.216
E E  4UWQA                119    0.546  95.474  -0.057   0.694   0.003   0.303
E A  4UWQA                120    0.601  66.186  -1.112   0.600   0.003   0.397
E E  4UWQA                121    0.605 105.641  -1.098   0.321   0.003   0.675
E G  4UWQA                122    0.434  34.156  -1.117   0.016   0.005   0.979
E G  4UWQA                123    0.332  26.160  -0.079   0.005   0.015   0.979
E K  4UWQA                124    0.322  66.277   0.452   0.004   0.197   0.799
B A  4UWQA                125    0.046   5.058   0.818   0.001   0.900   0.099
B L  4UWQA                126    0.022   3.955   1.270   0.001   0.959   0.040
B V  4UWQA                127    0.049   7.562   1.069   0.000   0.983   0.017
B L  4UWQA                128    0.020   3.625   0.960   0.001   0.959   0.040
B D  4UWQA                129    0.047   6.845  -0.716   0.003   0.718   0.279
B G  4UWQA                130    0.042   3.337  -1.759   0.004   0.138   0.858
B G  4UWQA                131    0.095   7.461  -1.821   0.018   0.019   0.964
B D  4UWQA                132    0.173  24.944  -1.488   0.018   0.047   0.935
B T  4UWQA                133    0.212  29.418  -0.618   0.019   0.141   0.840
B W  4UWQA                134    0.219  52.573  -0.332   0.019   0.141   0.840
E T  4UWQA                135    0.314  43.552  -1.590   0.019   0.141   0.840
B N  4UWQA                136    0.235  34.389  -1.835   0.018   0.088   0.893
B S  4UWQA                137    0.104  12.189  -1.065   0.053   0.043   0.903
B G  4UWQA                138    0.111   8.775  -1.870   0.354   0.048   0.598
B L  4UWQA                139    0.117  21.478  -0.403   0.502   0.102   0.396
B S  4UWQA                140    0.088  10.290  -1.556   0.502   0.102   0.396
B L  4UWQA                141    0.203  37.114  -1.319   0.502   0.102   0.396
B L  4UWQA                142    0.177  32.427  -0.810   0.278   0.093   0.628
B T  4UWQA                143    0.199  27.601  -1.551   0.184   0.043   0.773
E R  4UWQA                144    0.371  85.028   0.392   0.181   0.016   0.803
B G  4UWQA                145    0.202  15.929  -1.184   0.257   0.016   0.727
E E  4UWQA                146    0.448  78.335   0.979   0.717   0.014   0.269
B A  4UWQA                147    0.234  25.831  -0.105   0.879   0.010   0.111
B V  4UWQA                148    0.052   7.962  -0.016   0.938   0.007   0.055
B V  4UWQA                149    0.057   8.715   1.005   0.923   0.002   0.076
E R  4UWQA                150    0.436  99.752   1.990   0.970   0.001   0.030
B W  4UWQA                151    0.050  12.025   0.053   0.970   0.001   0.030
B Q  4UWQA                152    0.056   9.948   0.189   0.923   0.002   0.076
E N  4UWQA                153    0.387  56.730   0.358   0.858   0.002   0.139
E L  4UWQA                154    0.438  80.179  -0.386   0.782   0.003   0.216
B V  4UWQA                155    0.065  10.037   0.160   0.406   0.004   0.590
E G  4UWQA                156    0.304  23.909  -0.309   0.016   0.005   0.979
B V  4UWQA                157    0.067  10.359   0.235   0.018   0.047   0.935
B D  4UWQA                158    0.052   7.508  -1.010   0.021   0.279   0.699
B H  4UWQA                159    0.017   3.092   1.207   0.002   0.816   0.182
B M  4UWQA                160    0.019   3.802   0.904   0.011   0.918   0.071
B V  4UWQA                161    0.014   2.121   1.515   0.021   0.756   0.223
B S  4UWQA                162    0.023   2.742  -0.135   0.022   0.359   0.619
B H  4UWQA                163    0.048   8.749  -0.314   0.018   0.088   0.893
B C  4UWQA                164    0.075  10.474   0.671   0.056   0.142   0.802
B E  4UWQA                165    0.069  11.984  -0.858   0.118   0.150   0.732
B W  4UWQA                166    0.057  13.757   0.683   0.113   0.087   0.800
B T  4UWQA                167    0.217  30.153  -0.634   0.053   0.043   0.903
E L  4UWQA                168    0.275  50.316   0.609   0.058   0.017   0.925
B G  4UWQA                169    0.205  16.141  -0.963   0.058   0.017   0.925
E R  4UWQA                170    0.326  74.677   0.620   0.858   0.002   0.139
E E  4UWQA                171    0.687 120.036   0.993   0.923   0.002   0.076
B R  4UWQA                172    0.229  52.349   1.210   0.970   0.001   0.030
B V  4UWQA                173    0.016   2.505   1.427   0.970   0.001   0.030
E E  4UWQA                174    0.326  56.882   2.063   0.970   0.001   0.030
E E  4UWQA                175    0.525  91.805   1.885   0.970   0.001   0.030
B L  4UWQA                176    0.055  10.144   1.135   0.970   0.001   0.030
B L  4UWQA                177    0.064  11.682   0.284   0.923   0.002   0.076
E G  4UWQA                178    0.699  55.003   1.259   0.858   0.002   0.139
E L  4UWQA                179    0.480  87.961   0.512   0.694   0.003   0.303
B F  4UWQA                180    0.041   8.189  -0.749   0.246   0.004   0.750
E R  4UWQA                181    0.700 160.277   1.273   0.003   0.003   0.994
B G  4UWQA                182    0.140  11.026   0.513   0.005   0.015   0.979
E E  4UWQA                183    0.433  75.610   0.768   0.004   0.085   0.910
B F  4UWQA                184    0.089  17.943   0.727   0.002   0.816   0.182
B L  4UWQA                185    0.042   7.654  -0.284   0.001   0.959   0.040
B S  4UWQA                186    0.029   3.364  -0.647   0.018   0.846   0.136
B Y  4UWQA                187    0.039   8.270  -1.476   0.021   0.756   0.223
B N  4UWQA                188    0.071  10.350  -0.983   0.022   0.552   0.426
B I  4UWQA                189    0.071  13.227   0.596   0.022   0.552   0.426
B V  4UWQA                190    0.219  33.660   0.595   0.022   0.552   0.426
E D  4UWQA                191    0.298  42.899   0.695   0.022   0.552   0.426
E D  4UWQA                192    0.558  80.336  -0.125   0.064   0.216   0.721
E L  4UWQA                193    0.673 123.190  -0.669   0.053   0.043   0.903
E F  4UWQA                194    0.602 120.862  -0.992   0.058   0.017   0.925
E G  4UWQA                195    0.523  41.152  -1.350   0.018   0.019   0.964
E D  4UWQA                196    0.447  64.413   0.037   0.018   0.047   0.935
E P  4UWQA                197    0.457  64.905  -0.470   0.019   0.141   0.840
E L  4UWQA                198    0.334  61.100  -0.170   0.021   0.279   0.699
B F  4UWQA                199    0.088  17.601  -0.209   0.005   0.336   0.660
E P  4UWQA                200    0.490  69.559   0.782   0.004   0.197   0.799
E A  4UWQA                201    0.314  34.658   0.421   0.005   0.336   0.660
B Y  4UWQA                202    0.091  19.447   0.595   0.001   0.900   0.099
B R  4UWQA                203    0.144  32.907   1.076   0.001   0.959   0.040
B I  4UWQA                204    0.103  18.999   0.554   0.000   0.983   0.017
B H  4UWQA                205    0.155  28.249   1.373   0.001   0.959   0.040
E R  4UWQA                206    0.341  78.158   0.211   0.001   0.959   0.040
E V  4UWQA                207    0.238  36.519   0.608   0.004   0.616   0.381
E G  4UWQA                208    0.732  57.585  -0.151   0.005   0.015   0.979
E P  4UWQA                209    0.759 107.773  -1.015   0.003   0.003   0.994
B Y  4UWQA                210    0.168  35.795   0.496   0.003   0.718   0.279
E A  4UWQA                211    0.336  37.038   1.433   0.001   0.900   0.099
B L  4UWQA                212    0.036   6.573   0.611   0.001   0.959   0.040
B A  4UWQA                213    0.035   3.857  -0.024   0.001   0.959   0.040
B V  4UWQA                214    0.022   3.351   0.777   0.001   0.959   0.040
B V  4UWQA                215    0.023   3.597   1.020   0.001   0.959   0.040
B G  4UWQA                216    0.021   1.621  -0.116   0.001   0.900   0.099
B A  4UWQA                217    0.041   4.474   0.051   0.002   0.816   0.182
B S  4UWQA                218    0.039   4.629  -0.099   0.004   0.616   0.381
B Y  4UWQA                219    0.097  20.665   0.394   0.021   0.279   0.699
E P  4UWQA                220    0.440  62.450  -0.652   0.058   0.017   0.925
E Y  4UWQA                221    0.334  71.397  -0.919   0.115   0.016   0.868
B V  4UWQA                222    0.138  21.180  -0.392   0.113   0.043   0.844
E K  4UWQA                223    0.381  78.475  -1.238   0.113   0.043   0.844
E V  4UWQA                224    0.424  65.092  -1.047   0.113   0.087   0.800
B S  4UWQA                225    0.199  23.311  -0.666   0.113   0.087   0.800
E H  4UWQA                226    0.391  71.214  -1.103   0.053   0.043   0.903
E P  4UWQA                227    0.369  52.319  -0.520   0.058   0.017   0.925
E E  4UWQA                228    0.620 108.349  -1.238   0.058   0.017   0.925
E S  4UWQA                229    0.644  75.512  -0.794   0.058   0.017   0.925
E F  4UWQA                230    0.298  59.789  -0.285   0.053   0.043   0.903
B T  4UWQA                231    0.220  30.556  -0.875   0.018   0.047   0.935
E E  4UWQA                232    0.618 108.034  -0.774   0.018   0.047   0.935
E G  4UWQA                233    0.492  38.705   0.129   0.018   0.088   0.893
B L  4UWQA                234    0.051   9.393   0.752   0.022   0.552   0.426
E S  4UWQA                235    0.459  53.783   1.219   0.003   0.718   0.279
B F  4UWQA                236    0.111  22.338  -0.506   0.003   0.718   0.279
E A  4UWQA                237    0.389  42.912   0.573   0.021   0.451   0.528
E L  4UWQA                238    0.442  80.967  -0.064   0.113   0.087   0.800
B D  4UWQA                239    0.197  28.417   0.490   0.923   0.002   0.076
B E  4UWQA                240    0.129  22.484   0.603   0.970   0.001   0.030
E R  4UWQA                241    0.522 119.538   1.456   0.970   0.001   0.030
B R  4UWQA                242    0.139  31.739   1.200   0.970   0.001   0.030
B L  4UWQA                243    0.016   2.930   1.652   0.970   0.001   0.030
E Q  4UWQA                244    0.369  65.939   2.344   0.970   0.001   0.030
E E  4UWQA                245    0.516  90.163   1.949   0.970   0.001   0.030
B A  4UWQA                246    0.072   7.967   0.851   0.970   0.001   0.030
B V  4UWQA                247    0.022   3.335   1.230   0.970   0.001   0.030
E D  4UWQA                248    0.542  78.117   2.223   0.970   0.001   0.030
E K  4UWQA                249    0.427  87.752   2.372   0.970   0.001   0.030
B A  4UWQA                250    0.029   3.141   0.660   0.923   0.002   0.076
E R  4UWQA                251    0.386  88.279   2.091   0.923   0.002   0.076
E A  4UWQA                252    0.802  88.402   1.675   0.694   0.003   0.303
E E  4UWQA                253    0.509  88.905   1.798   0.176   0.004   0.820
E G  4UWQA                254    0.613  48.282   0.639   0.003   0.003   0.994
B A  4UWQA                255    0.036   4.000   0.969   0.005   0.015   0.979
E N  4UWQA                256    0.384  56.262   1.697   0.004   0.138   0.858
B A  4UWQA                257    0.051   5.598   1.496   0.001   0.959   0.040
B V  4UWQA                258    0.025   3.766   1.350   0.000   0.983   0.017
B V  4UWQA                259    0.013   1.952   2.042   0.000   0.983   0.017
B L  4UWQA                260    0.014   2.582   1.601   0.000   0.983   0.017
B L  4UWQA                261    0.018   3.259   1.403   0.001   0.900   0.099
B S  4UWQA                262    0.038   4.395  -0.331   0.004   0.616   0.381
B H  4UWQA                263    0.060  10.950  -1.493   0.005   0.262   0.733
B N  4UWQA                264    0.100  14.713  -0.178   0.018   0.047   0.935
E G  4UWQA                265    0.206  16.189  -1.317   0.018   0.019   0.964
B M  4UWQA                266    0.245  49.045  -0.266   0.339   0.016   0.645
E Q  4UWQA                267    0.530  94.640  -1.259   0.600   0.003   0.397
E L  4UWQA                268    0.317  58.043  -0.168   0.600   0.003   0.397
B D  4UWQA                269    0.082  11.802  -1.037   0.782   0.003   0.216
E A  4UWQA                270    0.343  37.821   1.564   0.923   0.002   0.076
E A  4UWQA                271    0.481  53.039   1.697   0.970   0.001   0.030
B L  4UWQA                272    0.043   7.892   0.516   0.970   0.001   0.030
B A  4UWQA                273    0.045   4.926  -0.040   0.970   0.001   0.030
E E  4UWQA                274    0.647 113.013   1.696   0.923   0.002   0.076
E R  4UWQA                275    0.472 108.111   1.782   0.782   0.003   0.216
B I  4UWQA                276    0.044   8.066   0.644   0.321   0.003   0.675
E R  4UWQA                277    0.660 151.209   0.904   0.016   0.005   0.979
E G  4UWQA                278    0.503  39.594   0.344   0.005   0.015   0.979
B I  4UWQA                279    0.032   5.883   1.105   0.004   0.138   0.858
B D  4UWQA                280    0.183  26.370   0.865   0.004   0.420   0.576
B L  4UWQA                281    0.026   4.816   1.149   0.001   0.959   0.040
B I  4UWQA                282    0.019   3.552   1.270   0.001   0.959   0.040
B L  4UWQA                283    0.014   2.637   1.501   0.001   0.900   0.099
B S  4UWQA                284    0.021   2.485   0.120   0.004   0.616   0.381
B G  4UWQA                285    0.029   2.282  -0.536   0.004   0.197   0.799
B H  4UWQA                286    0.061  11.041  -0.369   0.004   0.085   0.910
B T  4UWQA                287    0.079  10.888  -0.713   0.018   0.047   0.935
B H  4UWQA                288    0.037   6.712  -0.662   0.053   0.043   0.903
B D  4UWQA                289    0.202  29.094   0.190   0.053   0.043   0.903
B L  4UWQA                290    0.210  38.396  -0.780   0.052   0.084   0.864
B T  4UWQA                291    0.133  18.433   0.296   0.019   0.141   0.840
E P  4UWQA                292    0.468  66.409  -1.421   0.020   0.205   0.775
E R  4UWQA                293    0.484 110.859  -0.783   0.019   0.141   0.840
E P  4UWQA                294    0.429  60.861  -1.616   0.019   0.141   0.840
E W  4UWQA                295    0.355  85.257  -1.105   0.022   0.359   0.619
E R  4UWQA                296    0.413  94.577  -0.555   0.021   0.451   0.528
E V  4UWQA                297    0.432  66.337  -1.749   0.021   0.279   0.699
E G  4UWQA                298    0.684  53.791  -1.772   0.005   0.045   0.951
E K  4UWQA                299    0.513 105.565  -0.232   0.004   0.085   0.910
B T  4UWQA                300    0.097  13.496   0.018   0.003   0.718   0.279
B W  4UWQA                301    0.140  33.766   0.698   0.001   0.900   0.099
B I  4UWQA                302    0.043   7.973  -0.589   0.001   0.959   0.040
B V  4UWQA                303    0.027   4.088  -0.042   0.001   0.900   0.099
B A  4UWQA                304    0.114  12.519  -0.168   0.002   0.816   0.182
B G  4UWQA                305    0.048   3.809  -2.246   0.004   0.514   0.481
B S  4UWQA                306    0.192  22.538  -1.893   0.021   0.279   0.699
E A  4UWQA                307    0.369  40.653  -1.490   0.064   0.216   0.721
B A  4UWQA                308    0.259  28.542  -0.395   0.191   0.086   0.723
B G  4UWQA                309    0.075   5.871  -2.534   0.307   0.165   0.527
E K  4UWQA                310    0.376  77.425   0.811   0.252   0.423   0.325
B A  4UWQA                311    0.146  16.078  -0.019   0.087   0.683   0.230
B L  4UWQA                312    0.073  13.403  -0.129   0.085   0.775   0.140
B M  4UWQA                313    0.077  15.308  -1.221   0.011   0.918   0.071
B R  4UWQA                314    0.141  32.266   0.375   0.006   0.962   0.032
B V  4UWQA                315    0.038   5.902  -0.522   0.001   0.959   0.040
B D  4UWQA                316    0.154  22.249   0.347   0.006   0.962   0.032
B L  4UWQA                317    0.075  13.806  -0.713   0.006   0.962   0.032
E K  4UWQA                318    0.264  54.346   0.249   0.011   0.918   0.071
B L  4UWQA                319    0.137  25.085   0.057   0.018   0.846   0.136
E W  4UWQA                320    0.382  91.775  -0.353   0.021   0.756   0.223
E K  4UWQA                321    0.386  79.421  -0.887   0.021   0.279   0.699
E G  4UWQA                322    0.639  50.289  -1.450   0.018   0.088   0.893
E G  4UWQA                323    0.564  44.379  -0.834   0.052   0.084   0.864
E I  4UWQA                324    0.401  74.167  -0.666   0.066   0.296   0.638
E A  4UWQA                325    0.333  36.675  -0.467   0.022   0.552   0.426
E N  4UWQA                326    0.318  46.526  -0.335   0.023   0.655   0.322
B L  4UWQA                327    0.272  49.876  -0.188   0.023   0.655   0.322
B R  4UWQA                328    0.273  62.471  -0.410   0.022   0.552   0.426
B V  4UWQA                329    0.261  40.054  -1.874   0.021   0.451   0.528
E R  4UWQA                330    0.346  79.280  -0.933   0.022   0.552   0.426
B V  4UWQA                331    0.204  31.339  -0.509   0.074   0.484   0.442
B L  4UWQA                332    0.219  40.172  -0.477   0.069   0.386   0.545
E P  4UWQA                333    0.340  48.189  -0.521   0.135   0.317   0.548
E V  4UWQA                334    0.401  61.618  -0.873   0.125   0.227   0.648
E L  4UWQA                335    0.316  57.786  -1.003   0.199   0.152   0.649
E A  4UWQA                336    0.402  44.344  -1.208   0.191   0.086   0.723
E E  4UWQA                337    0.543  94.897  -1.508   0.278   0.093   0.628
E H  4UWQA                338    0.439  79.927  -0.688   0.199   0.152   0.649
B L  4UWQA                339    0.237  43.376  -0.631   0.199   0.152   0.649
E P  4UWQA                340    0.406  57.583  -1.227   0.199   0.152   0.649
E K  4UWQA                341    0.365  75.163  -0.581   0.191   0.086   0.723
B A  4UWQA                342    0.257  28.299  -0.582   0.181   0.016   0.803
E E  4UWQA                343    0.567  99.072  -0.581   0.782   0.003   0.216
E D  4UWQA                344    0.462  66.531  -0.017   0.858   0.002   0.139
B V  4UWQA                345    0.059   9.130  -0.156   0.923   0.002   0.076
B E  4UWQA                346    0.237  41.334   0.376   0.923   0.002   0.076
E A  4UWQA                347    0.492  54.229   0.836   0.858   0.002   0.139
B F  4UWQA                348    0.127  25.509   0.112   0.858   0.002   0.139
B L  4UWQA                349    0.041   7.544  -0.003   0.858   0.002   0.139
E K  4UWQA                350    0.452  92.894   1.120   0.923   0.002   0.076
E A  4UWQA                351    0.454  50.042   1.270   0.923   0.002   0.076
B Q  4UWQA                352    0.055   9.787   0.595   0.923   0.002   0.076
B L  4UWQA                353    0.246  45.006   0.621   0.938   0.007   0.055
E A  4UWQA                354    0.518  57.117   0.431   0.938   0.007   0.055
E P  4UWQA                355    0.347  49.168   1.207   0.879   0.010   0.111
B H  4UWQA                356    0.052   9.404  -0.436   0.858   0.002   0.139
E Q  4UWQA                357    0.393  70.243   0.564   0.879   0.010   0.111
E D  4UWQA                358    0.668  96.201   0.456   0.879   0.010   0.111
B H  4UWQA                359    0.256  46.475   0.046   0.717   0.014   0.269
B L  4UWQA                360    0.251  45.976  -0.612   0.522   0.016   0.462
E F  4UWQA                361    0.684 137.279  -0.182   0.113   0.043   0.844
E T  4UWQA                362    0.407  56.451   0.766   0.021   0.279   0.699
E P  4UWQA                363    0.262  37.149   0.394   0.021   0.756   0.223
B L  4UWQA                364    0.090  16.424   0.851   0.011   0.918   0.071
B A  4UWQA                365    0.070   7.725  -1.160   0.011   0.918   0.071
E V  4UWQA                366    0.337  51.751   0.887   0.011   0.918   0.071
B S  4UWQA                367    0.062   7.231  -0.068   0.023   0.655   0.322
E E  4UWQA                368    0.448  78.248  -0.202   0.021   0.451   0.528
B T  4UWQA                369    0.265  36.783  -0.660   0.021   0.451   0.528
E L  4UWQA                370    0.347  63.481  -0.475   0.069   0.386   0.545
B L  4UWQA                371    0.073  13.311  -1.418   0.125   0.227   0.648
E Y  4UWQA                372    0.327  69.965  -0.674   0.118   0.150   0.732
E K  4UWQA                373    0.382  78.577  -1.640   0.113   0.043   0.844
E R  4UWQA                374    0.495 113.378  -1.877   0.113   0.043   0.844
E D  4UWQA                375    0.333  48.029  -1.068   0.113   0.043   0.844
B T  4UWQA                376    0.265  36.769  -1.560   0.113   0.043   0.844
E L  4UWQA                377    0.329  60.222  -1.045   0.053   0.043   0.903
B Y  4UWQA                378    0.233  49.835  -1.776   0.058   0.017   0.925
B S  4UWQA                379    0.079   9.306  -0.831   0.018   0.019   0.964
B T  4UWQA                380    0.187  25.937  -1.158   0.181   0.016   0.803
B W  4UWQA                381    0.062  15.031  -0.371   0.622   0.015   0.363
B D  4UWQA                382    0.048   6.974  -0.580   0.858   0.002   0.139
B Q  4UWQA                383    0.181  32.255  -0.151   0.923   0.002   0.076
B L  4UWQA                384    0.038   7.049  -0.247   0.970   0.001   0.030
B V  4UWQA                385    0.026   4.058   0.644   0.970   0.001   0.030
B G  4UWQA                386    0.035   2.778  -0.305   0.970   0.001   0.030
B E  4UWQA                387    0.172  30.118   0.095   0.970   0.001   0.030
B A  4UWQA                388    0.097  10.700   0.088   0.970   0.001   0.030
B V  4UWQA                389    0.077  11.758  -0.141   0.970   0.001   0.030
E K  4UWQA                390    0.247  50.787   0.736   0.970   0.001   0.030
E A  4UWQA                391    0.436  48.014  -0.089   0.923   0.002   0.076
B I  4UWQA                392    0.184  34.003   0.039   0.858   0.002   0.139
B Y  4UWQA                393    0.251  53.553  -0.343   0.522   0.016   0.462
B P  4UWQA                394    0.196  27.741   0.379   0.115   0.016   0.868
E E  4UWQA                395    0.394  68.867   0.604   0.018   0.088   0.893
B V  4UWQA                396    0.017   2.644   1.395   0.021   0.451   0.528
B E  4UWQA                397    0.044   7.617  -0.952   0.023   0.655   0.322
B V  4UWQA                398    0.018   2.828   1.153   0.018   0.846   0.136
B V  4UWQA                399    0.022   3.320   0.372   0.018   0.846   0.136
B F  4UWQA                400    0.026   5.319  -0.174   0.022   0.552   0.426
B S  4UWQA                401    0.056   6.575  -0.458   0.020   0.205   0.775
B P  4UWQA                402    0.059   8.386  -2.462   0.018   0.047   0.935
B A  4UWQA                403    0.038   4.210  -3.334   0.018   0.088   0.893
B V  4UWQA                404    0.070  10.774  -2.653   0.020   0.205   0.775
B R  4UWQA                405    0.146  33.342  -1.622   0.020   0.205   0.775
B W  4UWQA                406    0.185  44.589  -1.752   0.052   0.084   0.864
E G  4UWQA                407    0.335  26.341  -1.852   0.018   0.047   0.935
E T  4UWQA                408    0.355  49.280  -1.286   0.018   0.019   0.964
E T  4UWQA                409    0.529  73.317  -1.854   0.053   0.043   0.903
E I  4UWQA                410    0.413  76.460  -1.003   0.053   0.043   0.903
B L  4UWQA                411    0.161  29.479  -0.917   0.018   0.047   0.935
E P  4UWQA                412    0.563  79.918  -1.169   0.018   0.019   0.964
E G  4UWQA                413    0.583  45.906  -1.291   0.058   0.017   0.925
E Q  4UWQA                414    0.268  47.776  -0.980   0.184   0.043   0.773
E A  4UWQA                415    0.410  45.127   0.709   0.386   0.097   0.517
B I  4UWQA                416    0.069  12.802   0.754   0.478   0.309   0.214
B T  4UWQA                417    0.146  20.181   0.743   0.453   0.248   0.299
B W  4UWQA                418    0.179  43.098   0.786   0.938   0.007   0.055
E D  4UWQA                419    0.361  52.078   0.670   0.938   0.007   0.055
B H  4UWQA                420    0.117  21.228   0.327   0.879   0.010   0.111
B L  4UWQA                421    0.027   4.962   0.285   0.911   0.033   0.057
B Y  4UWQA                422    0.151  32.333   0.538   0.911   0.033   0.057
E A  4UWQA                423    0.378  41.689   0.749   0.831   0.044   0.125
B Y  4UWQA                424    0.058  12.459  -0.360   0.660   0.049   0.291
B T  4UWQA                425    0.077  10.722   0.820   0.307   0.165   0.527
B G  4UWQA                426    0.213  16.755  -0.981   0.056   0.142   0.802
B F  4UWQA                427    0.122  24.546   0.032   0.018   0.088   0.893
E T  4UWQA                428    0.512  71.042  -1.336   0.018   0.088   0.893
B Y  4UWQA                429    0.232  49.643  -1.422   0.018   0.019   0.964
E P  4UWQA                430    0.329  46.713  -1.457   0.018   0.088   0.893
E E  4UWQA                431    0.298  52.096  -0.231   0.021   0.451   0.528
B L  4UWQA                432    0.098  17.907   0.174   0.011   0.918   0.071
B Y  4UWQA                433    0.088  18.763  -0.120   0.006   0.962   0.032
B L  4UWQA                434    0.168  30.706  -0.302   0.006   0.962   0.032
B F  4UWQA                435    0.058  11.641   0.488   0.001   0.959   0.040
E Y  4UWQA                436    0.349  74.539   0.673   0.001   0.900   0.099
B L  4UWQA                437    0.031   5.621   0.538   0.003   0.718   0.279
E R  4UWQA                438    0.261  59.838   1.420   0.005   0.262   0.733
B G  4UWQA                439    0.066   5.163  -0.414   0.879   0.010   0.111
E A  4UWQA                440    0.552  60.797   1.509   0.970   0.001   0.030
E Q  4UWQA                441    0.251  44.775   1.719   0.975   0.003   0.022
B I  4UWQA                442    0.012   2.257   1.780   0.975   0.003   0.022
B K  4UWQA                443    0.225  46.303   1.877   0.970   0.001   0.030
E A  4UWQA                444    0.440  48.466   1.823   0.970   0.001   0.030
B V  4UWQA                445    0.052   8.039   1.368   0.970   0.001   0.030
B L  4UWQA                446    0.028   5.182   0.827   0.970   0.001   0.030
E E  4UWQA                447    0.314  54.856   1.091   0.970   0.001   0.030
E D  4UWQA                448    0.321  46.285   0.545   0.923   0.002   0.076
B I  4UWQA                449    0.039   7.234  -1.003   0.858   0.002   0.139
B A  4UWQA                450    0.091  10.072  -0.165   0.782   0.003   0.216
E S  4UWQA                451    0.461  54.076   0.367   0.694   0.003   0.303
E N  4UWQA                452    0.317  46.438  -0.114   0.622   0.015   0.363
B V  4UWQA                453    0.183  28.081  -1.398   0.522   0.016   0.462
B F  4UWQA                454    0.309  61.936  -1.450   0.430   0.016   0.555
E T  4UWQA                455    0.477  66.104  -1.421   0.354   0.048   0.598
E S  4UWQA                456    0.560  65.585  -1.916   0.184   0.043   0.773
E D  4UWQA                457    0.475  68.390  -1.575   0.053   0.043   0.903
E P  4UWQA                458    0.396  56.207  -2.096   0.113   0.043   0.844
E F  4UWQA                459    0.335  67.194  -1.739   0.113   0.043   0.844
E Y  4UWQA                460    0.423  90.374  -1.387   0.053   0.043   0.903
E Q  4UWQA                461    0.411  73.333  -1.577   0.052   0.084   0.864
E Q  4UWQA                462    0.478  85.442  -1.714   0.053   0.043   0.903
E G  4UWQA                463    0.433  34.101  -2.591   0.018   0.047   0.935
B G  4UWQA                464    0.261  20.525  -2.395   0.018   0.047   0.935
E D  4UWQA                465    0.298  42.942  -1.638   0.018   0.088   0.893
B V  4UWQA                466    0.231  35.582  -0.728   0.005   0.336   0.660
B S  4UWQA                467    0.092  10.794  -0.242   0.004   0.514   0.481
B R  4UWQA                468    0.179  41.060  -0.466   0.002   0.816   0.182
B V  4UWQA                469    0.062   9.545  -1.599   0.001   0.900   0.099
B F  4UWQA                470    0.128  25.629  -1.855   0.003   0.718   0.279
B G  4UWQA                471    0.139  10.931  -2.432   0.004   0.514   0.481
B L  4UWQA                472    0.053   9.759   0.250   0.002   0.816   0.182
B R  4UWQA                473    0.192  43.854   0.954   0.001   0.959   0.040
B Y  4UWQA                474    0.064  13.634   0.607   0.001   0.959   0.040
B V  4UWQA                475    0.180  27.666  -0.009   0.006   0.962   0.032
B L  4UWQA                476    0.114  20.800   0.584   0.011   0.918   0.071
B D  4UWQA                477    0.186  26.803  -0.416   0.002   0.816   0.182
B P  4UWQA                478    0.227  32.197  -0.351   0.005   0.336   0.660
E D  4UWQA                479    0.687  98.997  -0.968   0.005   0.045   0.951
E A  4UWQA                480    0.372  40.972   0.390   0.005   0.015   0.979
E P  4UWQA                481    0.541  76.782  -0.751   0.018   0.019   0.964
E T  4UWQA                482    0.579  80.280  -0.686   0.018   0.019   0.964
E G  4UWQA                483    0.467  36.777  -1.149   0.018   0.047   0.935
E E  4UWQA                484    0.389  67.888  -0.331   0.004   0.138   0.858
E R  4UWQA                485    0.301  68.952   1.041   0.004   0.616   0.381
B V  4UWQA                486    0.045   6.963  -0.147   0.001   0.900   0.099
E R  4UWQA                487    0.250  57.136   0.942   0.001   0.900   0.099
E E  4UWQA                488    0.358  62.595   0.660   0.001   0.959   0.040
B V  4UWQA                489    0.047   7.239   0.877   0.001   0.959   0.040
E E  4UWQA                490    0.315  54.978   0.771   0.001   0.959   0.040
B V  4UWQA                491    0.077  11.804   0.037   0.001   0.959   0.040
E G  4UWQA                492    0.530  41.735   0.684   0.004   0.420   0.576
E G  4UWQA                493    0.612  48.196  -0.555   0.005   0.045   0.951
E R  4UWQA                494    0.535 122.538   1.590   0.003   0.718   0.279
E P  4UWQA                495    0.504  71.461   0.957   0.002   0.816   0.182
B L  4UWQA                496    0.086  15.692   1.132   0.005   0.336   0.660
E D  4UWQA                497    0.434  62.511   1.758   0.004   0.085   0.910
E P  4UWQA                498    0.506  71.830   0.474   0.005   0.045   0.951
E N  4UWQA                499    0.718 105.101   0.778   0.005   0.015   0.979
E R  4UWQA                500    0.329  75.226   1.477   0.004   0.197   0.799
E R  4UWQA                501    0.298  68.334   1.645   0.002   0.816   0.182
B Y  4UWQA                502    0.036   7.757   0.857   0.001   0.959   0.040
B L  4UWQA                503    0.198  36.217   1.616   0.001   0.959   0.040
B A  4UWQA                504    0.026   2.898   0.790   0.001   0.959   0.040
B A  4UWQA                505    0.020   2.204   0.569   0.001   0.959   0.040
B A  4UWQA                506    0.041   4.474  -1.072   0.002   0.816   0.182
B Y  4UWQA                507    0.193  41.308  -0.929   0.004   0.420   0.576
B G  4UWQA                508    0.232  18.227  -2.280   0.018   0.047   0.935
B G  4UWQA                509    0.307  24.153  -1.998   0.018   0.019   0.964
E R  4UWQA                510    0.298  68.150  -2.069   0.052   0.084   0.864
B L  4UWQA                511    0.137  25.011  -0.227   0.056   0.142   0.802
B Q  4UWQA                512    0.278  49.615  -1.455   0.052   0.084   0.864
E R  4UWQA                513    0.371  85.028  -1.346   0.113   0.087   0.800
B V  4UWQA                514    0.192  29.464  -1.756   0.056   0.142   0.802
E G  4UWQA                515    0.313  24.672  -1.642   0.056   0.142   0.802
E E  4UWQA                516    0.660 115.285  -0.846   0.052   0.084   0.864
E A  4UWQA                517    0.315  34.702  -1.867   0.053   0.043   0.903
E K  4UWQA                518    0.339  69.773   0.174   0.052   0.084   0.864
E P  4UWQA                519    0.456  64.650  -0.660   0.056   0.142   0.802
E G  4UWQA                520    0.311  24.507  -0.786   0.064   0.216   0.721
B Y  4UWQA                521    0.268  57.272  -1.257   0.020   0.205   0.775
E E  4UWQA                522    0.485  84.677  -0.887   0.018   0.088   0.893
E P  4UWQA                523    0.408  57.867  -1.346   0.053   0.043   0.903
E R  4UWQA                524    0.353  80.860  -0.401   0.257   0.016   0.727
E P  4UWQA                525    0.305  43.251   0.539   0.430   0.016   0.555
B I  4UWQA                526    0.044   8.122  -0.175   0.879   0.010   0.111
B Y  4UWQA                527    0.111  23.657   1.066   0.970   0.001   0.030
E E  4UWQA                528    0.348  60.743   1.057   0.970   0.001   0.030
B V  4UWQA                529    0.039   6.071   1.003   0.970   0.001   0.030
B L  4UWQA                530    0.016   2.985   1.684   0.988   0.000   0.012
B A  4UWQA                531    0.090   9.918   1.139   0.988   0.000   0.012
E E  4UWQA                532    0.449  78.493   1.825   0.988   0.000   0.012
B Y  4UWQA                533    0.100  21.477   1.475   0.970   0.001   0.030
B L  4UWQA                534    0.035   6.445  -0.709   0.970   0.001   0.030
E R  4UWQA                535    0.498 114.042   1.612   0.923   0.002   0.076
E S  4UWQA                536    0.831  97.358   1.569   0.694   0.003   0.303
E V  4UWQA                537    0.365  56.147   1.719   0.176   0.004   0.820
E G  4UWQA                538    0.496  39.004  -0.150   0.005   0.015   0.979
E R  4UWQA                539    0.470 107.538   0.324   0.004   0.138   0.858
B V  4UWQA                540    0.060   9.161  -0.593   0.004   0.420   0.576
E R  4UWQA                541    0.503 115.187   1.001   0.004   0.420   0.576
B V  4UWQA                542    0.200  30.679  -0.532   0.021   0.279   0.699
E R  4UWQA                543    0.534 122.332   0.450   0.018   0.088   0.893
E P  4UWQA                544    0.468  66.438  -0.570   0.018   0.088   0.893
E E  4UWQA                545    0.610 106.567  -1.028   0.005   0.015   0.979
E P  4UWQA                546    0.485  68.765  -0.877   0.005   0.015   0.979
E N  4UWQA                547    0.278  40.655   0.655   0.005   0.336   0.660
B V  4UWQA                548    0.055   8.453   0.511   0.001   0.900   0.099
E K  4UWQA                549    0.352  72.427   1.401   0.001   0.959   0.040
B V  4UWQA                550    0.174  26.728  -0.436   0.001   0.959   0.040
B I  4UWQA                551    0.071  13.098   0.736   0.002   0.816   0.182
E G  4UWQA                552    0.325  25.617  -1.118   0.004   0.420   0.576
E R  4UWQA                553    0.391  89.493  -0.184   0.004   0.197   0.799
E N  4UWQA                554    0.652  95.497  -2.033   0.018   0.088   0.893
E Y  4UWQA                555    0.345  73.705  -0.378   0.018   0.019   0.964
E R  4UWQA                556    0.671 153.682  -0.565   0.016   0.005   0.979
B L  4UWQA                557    0.256  46.874  -0.094   0.016   0.005   0.979
E P  4UWQA                558    0.361  51.198  -1.240   0.018   0.019   0.964
E E  4UWQA                559    0.560  97.832  -1.253   0.005   0.045   0.951
B V  4UWQA                560    0.246  37.779  -0.336   0.004   0.085   0.910
E T  4UWQA                561    0.577  80.058  -0.259   0.018   0.088   0.893
E G  4UWQA                562    0.884  69.539  -1.289   0.003   0.003   0.994

Explain the output.  Go back.
```

---
